# Biomechanical model of cerebral vascular dynamics and their effect on CSF dynamics

**DOI:** 10.1186/2045-8118-12-S1-P14

**Published:** 2015-09-18

**Authors:** Christine Goffin, Lukas Theisgen, Klaus Radermacher

**Affiliations:** 1 RWTH Aachen University, Germany

## 

With advancing age venous wall thickness increases going along with a loss of elastic properties, the arterioles curl and capillary density decreases leading to a reduction of cross sectional area [1]. Even in healthy individuals these changes influence vascular and CSF dynamics, as a reduction of total cerebral blood flow, aqueduct and cervical stroke volume are reported [2]. In NPH these dynamics seem to be altered in a different way, as aqueduct stroke volume [2] and ICP amplitude are increased and arteriovenous delay is drastically reduced compared to normal aging [3]. However it is not clear in what way the described vascular alterations influence the pressure propagation inside the vessels and impact CSF dynamics.

So far no biomechanical model exists that investigates the influence of macroscopic and microscopic changes of cerebral blood vessels. That is why we put a model up for discussion that simulates vascular pressure propagation and enables the investigation of altered vascular properties in the context of NPH.

A Matlab Simulink model was developed reproducing each vessel section by a distensible compartment. Therefore the cerebral vascular tree was divided into 13 sections from carotid artery to venous sinuses and the pulsatile carotid artery and sinus pressure were inputted as Fourier series. The cross sectional area was varied according to literature data and flow resistance was implemented taking into account the rheological characteristics of blood. The Windkessel function and relaxation properties of vascular walls were integrated by a Voigt model, enabling the variation of wall properties for each section individually. Due to the distensible vessel walls each section interacts with the CSF compartment and autoregulation was implemented by a simple proportional controller. After parameterisation mean pressure and pressure amplitude in the vessel sections showed good accordance with literature values [4].

We have proposed a model of vascular dynamics that is able to identify the impact of altered vascular wall properties and structural changes. Furthermore the effect of different arterial and venous input pressure profiles can be analysed. These parameter analyses are part of our ongoing research.
